# Mitochondrial Drp1 recognizes and induces excessive mPTP opening after hypoxia through BAX-PiC and LRRK2-HK2

**DOI:** 10.1038/s41419-021-04343-x

**Published:** 2021-11-05

**Authors:** Chenyang Duan, Lei Kuang, Chen Hong, Xinming Xiang, Jiancang Liu, Qinghui Li, Xiaoyong Peng, Yuanqun Zhou, Hongchen Wang, Liangming Liu, Tao Li

**Affiliations:** 1 State Key Laboratory of Trauma, Burns and Combined Injury, Shock and Transfusion Department, Research Institute of Surgery, Daping Hospital, Army Medical University, 400042 Chongqing, P.R. China; 2 Department of Anesthesiology, The Second Affiliated Hospital of Chongqing Medical University, 400010 Chongqing, P.R. China

**Keywords:** Apoptosis, Mitochondria

## Abstract

Mitochondrial mass imbalance is one of the key causes of cardiovascular dysfunction after hypoxia. The activation of dynamin-related protein 1 (Drp1), as well as its mitochondrial translocation, play important roles in the changes of both mitochondrial morphology and mitochondrial functions after hypoxia. However, in addition to mediating mitochondrial fission, whether Drp1 has other regulatory roles in mitochondrial homeostasis after mitochondrial translocation is unknown. In this study, we performed a series of interaction and colocalization assays and found that, after mitochondrial translocation, Drp1 may promote the excessive opening of the mitochondrial permeability transition pore (mPTP) after hypoxia. Firstly, mitochondrial Drp1 maximumly recognizes mPTP channels by binding Bcl-2-associated X protein (BAX) and a phosphate carrier protein (PiC) in the mPTP. Then, leucine-rich repeat serine/threonine-protein kinase 2 (LRRK2) is recruited, whose kinase activity is inhibited by direct binding with mitochondrial Drp1 after hypoxia. Subsequently, the mPTP-related protein hexokinase 2 (HK2) is inactivated at Thr-473 and dissociates from the mitochondrial membrane, ultimately causing structural disruption and overopening of mPTP, which aggravates mitochondrial and cellular dysfunction after hypoxia. Thus, our study interprets the dual direct regulation of mitochondrial Drp1 on mitochondrial morphology and functions after hypoxia and proposes a new mitochondrial fission-independent mechanism for the role of Drp1 after its translocation in hypoxic injury.

## Introduction

Hypoxic injury refers to tissue cell injury caused by insufficient blood perfusion and blood oxygen supply. It is a common pathway for the initiation and development of various critical illnesses, such as hemorrhagic shock, sepsis, and cardiopulmonary failure [1]. Severe hypoxic injury can cause organ dysfunction and may be life-threatening. The mitochondrion, as the main site of aerobic respiration in cells, is one of the organelles that undergo damage immediately after ischemia and hypoxia [2]. In our previous study, we found significant mitochondrial damage in tissues and organs, such as vascular [3], intestines [4], and the heart [5], after hypoxic injury. This mainly manifested as abnormal mitochondrial morphology (such as excessive fission, impaired fusion, etc.) and mitochondrial dysfunction (such as decreased ATP production, excessive ROS accumulation, etc.), which promoted organ dysfunction after hypoxia. However, the relationship between morphological and functional changes in the mitochondria following hypoxia has not been fully elucidated.

Dynamin-related protein 1 (Drp1), as a mitochondrial fission-associated GTPase, is a classical protein affecting mitochondrial morphological changes. Under normal conditions, free-state Drp1 molecules are located in the cytoplasm. After the hypoxic injury, activated Drp1 proteins translocate from cytoplasm to mitochondrial surface and cleaves mitochondrial phospholipid bilayer through its GTPase activity to facilitate mitochondrial fission, which then results in mitochondrial fragmentation [6]. However, whether Drp1 undergoing mitochondrial translocation has other roles in mitochondrial homeostasis except for regulating mitochondrial morphology after hypoxia is unknown.

The mitochondrial permeability transition pore (mPTP) is a non-selective, highly conductive composite channel that spans the inner and outer mitochondrial membranes and allows the passage of any molecule with a relative molecular mass lower than 1.5 kDa [7]. Excessive opening of mPTP results in mitochondrial expanding due to high osmotic pressure in the matrix, which leads to mitochondrial outer membrane rupture and mitochondrial morphology disruption [8]. Excessive opening of mPTP also causes reduced mitochondrial membrane potential and massive cytochrome C (CytC) release due to loss of ion flow selectivity, which then aggravates the accumulation of mitochondrial reactive oxygen species (ROS), ultimately leading to cell death [9]. Thus, excessive mPTP opening is considered a prerequisite for mitochondrial morphological and functional changes, but the specific regulatory mechanism is unknown. We have previously shown that activated Drp1 can regulate mitochondrial functions, such as ROS production and mitochondrial metabolism through a variety of mitochondrial fission-independent pathways after hypoxic injury [3, 4], whether mitochondrial Drp1 directly regulates the opening of mPTP channels has not been reported yet.

In this study, we investigated the direct regulatory mechanisms of mitochondrial Drp1 on mPTP opening after hypoxic injury and explored its links with classical Drp1 mitochondrial fission-dependent pathway. Our study clarified the close relationship between mitochondrial morphology and mitochondrial functions after hypoxia and interpreted the central position of the Drp1–mPTP pathway in the dual regulation of mitochondrial morphology and mitochondrial functions after hypoxia.

## Materials and methods

### Materials

Antibodies for Drp1 (Cat. ab56788), CytC (Cat. ab133504), BAX (Cat. ab32503), PiC (SLC25A3, Cat. ab89117), CK (Cat.ab108388), ATPase (Cat. ab76020), VDAC (Cat. ab154856), LRRK2 (Cat. ab133474) were purchased from Abcam (Cambridge, MA, USA). β-actin (Cat. ab8226), Tubulin (Cat. ab7291) and COX IV (Cat. ab202554) were used as interior references of total, cytoplasmic and mitochondrial fraction, which were also purchased from Abcam (Cambridge, MA, USA). Antibodies for ANT (Cat. PA1-85116) and CypD (Cat.PA5-31061) were purchased from Thermo (Waltham, MA, USA). Antibody for HK2 (Cat. 2106S) was purchased from Cell Signaling Technology (Danvers, MA, USA). Antibody for phospho-HK2 (Thr473) (Cat. 40343) was purchased from Abclonal Technology (Wuhan, China). MitoTracker Deep Red (Cat. M22426) was purchased from Invitrogen (Carlsbad, CA, USA). MPTP Assay Kit (Cat. C2009S), Calcein AM (Cat. C2012) were purchased from Beyotime Biotechnology (Shanghai, China). Adenoviral vectors for cytoplasmic Drp1 over-expression (Drp1 OE), Drp1 (T595A) mutation, HK2 (T473D) mutation were generated by Genechem Technology (Shanghai, China). Related activators and inhibitors including Mdivi-1 (Cat. S7162), CsA (Cat. S2286), BAI1 (Cat. S8865), NEM (Cat. S3692), BTSA1 (Cat. S8650), and SEW2871 (Cat. S7180) were purchased from Selleck (Shanghai, China). The Protein A/G Magnetic Beads IP Kit (Cat. 26162) was purchased from Thermo Scientific (Waltham, MA, USA) and fetal bovine serum (FBS) (Cat.10099141), as well as penicillin/streptomycin (Cat.10378016), were purchased from Invitrogen (Carlsbad, CA, USA). All other chemicals were purchased from Sigma unless specifically mentioned otherwise.

### Cell culture and hypoxia treatment

Vascular smooth muscle cells (VSMCs) were obtained from the superior mesenteric arteries (SMAs) of SD rats using an explant technique, as previously described [10]. VSMCs were cultured in Dulbecco-modified Eagle medium (DMEM)-F12 (Gibco, NY, USA) supplemented with 10% FBS (Hyclone, Logan, UT, USA) and 1% antibiotics. The third-to-fifth passage cells were used in the present study.

The hypoxia treatment was undertaken according to a previously reported protocol [10]. In brief, VSMCs were put into the hypoxia compartment (Ruskinn Bugbox, UK), bubbled with hypoxic gas (1% O_2_–5% –94% N_2_) at 3 L/min for 3 h, and then reoxygenated in normoxia condition (O_2_ concentration ~20%) for 4 h.

### mPTP opening detection

The opening of the mPTP was determined by the Calcein-CoCl_2_ staining method using confocal microscopy according to a previously reported protocol [11]. In brief, the treated cells were incubated with 2 μM Calcein and 100 nM MitoTracker Deep Red for 30 min in the absence of light. The cells were subsequently washed twice with PBS and then exposed to 2 mM CoCl_2_ for 15 min. The Calcein fluorescence is compartmentalized within the mitochondria until the mPTP opening permits the distribution of cobalt inside the mitochondria, which results in the quenching of Calcein fluorescence in the mitochondrial matrix. After three washes with PBS, fluorescence imaging of cells was performed with excitation at 488 nm (Calcein) or 633 nm (MitoTracker) and emission at 510–550 nm (Calcein) or 560–617 nm (MitoTracker) using the Leica TCS software (Leica Microsystems, Wetzlar, Germany). Digital images were analyzed using the Image J software (National Institutes of Health, Bethesda, MD, USA), and the degree of MPTP opening was reflected by the red (MitoTracker)/green (Calcein) fluorescence ratio.

### Subcellular fractionation

Isolated SMAs or VSMCs were collected in filter cartridges. The cytosol fractions were isolated using a Minute^TM^ Cytoplasmic Extraction Kit (Invent Biotechnologies, Inc. SC-003), and the mitochondria fractions were isolated using the Minute^TM^ Mitochondria Isolation Kit (Invent Biotechnologies, Inc. MP-007) [12]. Fractioned proteins were used for immunoblotting analyses with the indicated antibodies.

### Co-immunoprecipitation (Co-IP)

Co-IP was performed using the Protein A/G Magnetic Beads IP Kit according to the manufacturer’s instructions. 10 μg primary antibody was diluted with 200 μl PBST in a tube. 50 μl Protein A/G Magnetic Beads was added into the mixture and then incubated for 10 min at room temperature, and the antibody-conjugated immunomagnetic beads were prepared after removing the supernatant on the magnetic separator. After the samples were harvested, lysed and centrifuged, the supernatants were gently mixed with antibody-conjugated immunomagnetic beads to prepare an immunomagnetic beads–antibody–antigen complex. After washing the beads with PBS three times, the above complex was resuspended in 100 μl PBS and was used to detect endogenous interaction between proteins.

### Western blotting

Cell pellets were solubilized with the RIPA buffer (Beyotime Institute of Biotechnology, China) with the addition of cOmplete Protease Inhibitors (Roche, Switzerland) and PhosSTOP Phosphastase Inhibitors (Roche, Switzerland), electrophoresed, and blotted onto polyvinylidene fluoride membranes. The membranes were incubated with the indicated primary antibodies, followed by the incubation with horseradish peroxidase-conjugated secondary antibodies (Jackson ImmunoResearch, UK). The protein concentration was determined with the BCA Protein assay kit (Thermo Scientific Pierce, UK). Blotted proteins were visualized using an enhanced chemiluminescence detection kit (Tiangen Biotech, China). The intensity of the bands was analyzed by Quantity One V 4.62 software (Bio-Rad, Life Science, CA, USA) [3].

### Immunofluorescence staining

VSMCs were plated in the confocal chamber and incubated with MitoTracker Deep Red (100 nM for 30 min, 37 °C). After washing twice in 1× phosphate-buffered saline (PBS), VSMCs were fixed in a 4% paraformaldehyde solution for 20 min at room temperature. Cells were permeabilized with 0.1% Triton-x 100 in 1× PBS for 5 min at room temperature. Cells were then blocked in a 5% BSA solution for 1 h at room temperature, washed and incubated overnight at 4 °C with primary antibodies. Cells were then washed in PBS plus 0.1% Tween-20 and incubated with corresponding fluorophore-conjugated mouse or rabbit secondary antibodies (Invitrogen, Carlsbad, CA, USA) for 1 h at room temperature. Cells were washed as before with a final wash in 1× PBS alone and incubated with DAPI (BD Biosciences, Franklin Lakes, NJ, USA) (1:50) for 5 min at room temperature. Immunofluorescence was visualized using confocal laser-scanning microscopy (Leica SP5, Germany) [10].

### Proteome microarray detection

Biotin-tagged Drp1 was synthesized by KMD Bioscience (Tianjin, China). Arrayit HuProt™ v2.0 19K Human Proteome Microarrays (CDI Laboratories, Baltimore, MD, USA) were used to identify Drp1-interacting proteins. Microarrays were blocked with blocking buffer (1% BSA in 0.1% Tween 20; TBST) for 1 h at room temperature with gentle agitation. 50 µM biotin-tagged Drp1 or free biotin was added and incubated on the proteome microarray at room temperature for 1 h. The microarrays were washed with TBST three times for 5 min each washing and were incubated with Cy3-Streptavidin (1:1000, Sigma, St Louis, MO, USA) for 1 h at room temperature, followed by three 5-min washes in TBST. The microarrays were spun dry at 250 × *g* for 3 min and were scanned with a GenePix 4200A microarray scanner (Axon Instruments) to visualize and record the results [13]. The signal-to-noise ratio (SNR) was defined as the ratio of the median of the foreground signal to the median of the background signal and was calculated for each protein. The SNR of a protein was averaged from the two duplicated spots on each microarray and the mean SNR was used to represent the signal of the protein. To call the candidates, the cutoff of SNR was set as a ratio >3.0. Data were analyzed by GenePix Pro 6.0.

### Homology modeling and protein docking

The three-dimensional structures of LRRK2 and DRP1 proteins were constructed with the SWISS-MODEL web server (https://swissmodel.expasy.org/) using the homology modeling approach. A truncated sequence of LRRK2 protein from G1900 to G2090 was used due to the great length of this protein. The GRAMM-X Protein–Protein Docking web server was utilized to predict the complex structure of LRRK2 and DRP1 proteins. LRRK2 was defined as a ligand due to its smaller size, whereas DRP1 was defined as a receptor, and at most 10 models were output. The first of the three output models approximated the experimental result with the best outcome and was thus used as the final model. Since the GRAMM-X models were coarse, and unfavorable atomic clashes were found between receptor and ligand, the model was further minimized in UCSF Chimera with additional hydrogens and an AMBER ff14SB force field. Finally, PyMOL was used for visual analysis.

### Statistical analysis

Data are expressed as means and standard deviations. For each set of experiments, the sample size was chosen to ensure adequate power to detect variations. An independent sample *t*-test was used for experiments with two groups. One-way analysis of variance (ANOVA) was used for experiments with more than two groups and followed by Tukey’s post hoc analysis and SNK or LSD comparison using the SPSS 17.0 software (SPSS Inc., Chicago, IL, USA). *P* < 0.05 was considered to indicate a statistically significant difference in all analyses.

## Results

### Mitochondrial Drp1 affects mPTP opening after hypoxia

**Fig. 1:**
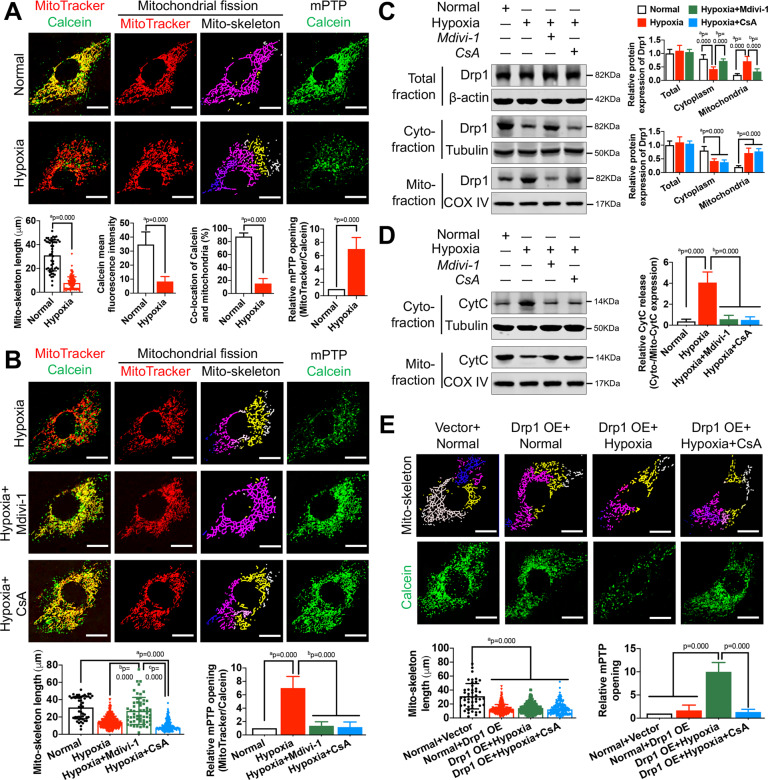
The effects of mitochondrial Drp1 on mPTP opening after hypoxia. **A** Representative confocal images of mPTP opening in VSMCs after hypoxia (bar, 25 μm). VSMCs were labeled with Calcein and MitoTracker to reflect mPTP opening and mitochondrial skeleton. **B** Representative confocal images of mPTP opening in hypoxia-induced VSMCs after Mdivi-1 (50 μM) or CsA (10 μM) treatment. Mitochondrial length and relative mPTP opening ratio in each group were calculated by Image J. **C** Relative protein expression of Drp1 in total, cytoplasmic and mitochondrial fractions of hypoxia-induced VSMCs after Mdivi-1 (50 μM) or CsA (10 μM) treatment. β-actin, Tubulin and COX IV were used as interior references of total, cytoplasmic and mitochondrial fractions. (*n* = 8 samples/group). **D** Relative protein expression of CytC in cytoplasmic and mitochondrial fractions of hypoxia-induced VSMCs after Mdivi-1 (50 μM) or CsA (10 μM) treatment. (*n* = 8 samples/group). **E** Representative confocal images of mPTP opening in Drp1-over expressed (Drp1 OE) VSMCs after hypoxia and CsA (10 μM) treatment. Mitochondrial length and relative mPTP opening ratio in each group were calculated by Image J. a: *P* < 0.05 compared with Normal group, b: *P* < 0.05 compared with Hypoxia group, c: *P* < 0.05 compared with Hypoxia + Mdivi-1 group.

In our previous study, we have shown that in vascular tissue injured by ischemia and hypoxia, Drp1 largely translocated from the cytoplasm to mitochondria where it mediated mitochondrial fission and affected mitochondrial morphology [3]. Here, we aimed to determine whether mitochondrial Drp1 has other biological roles in addition to mediating mitochondrial fission after hypoxia. We performed MitoTracker and Calcein fluorescence labeling to monitor mitochondrial morphological changes and mPTP opening in hypoxia-induced VSMC. The confocal images showed that both excessive mPTP opening (as indicated by an increase in mitochondrial Calcein fluorescence quenching) and excessive mitochondrial fission (as indicated by a decrease in mitochondrial skeleton length) were observed after hypoxia (Fig. 1A).

To determine whether mPTP overopening is closed associated with Drp1 undergoing mitochondrial translocation after hypoxia. we reduced mitochondrial Drp1 levels using Mdivi-1 (50 μM), an inhibitor of Drp1 translocation [3] and found that the mitochondrial skeleton length and Calcein fluorescence intensity were respectively 2.19-fold and 7.28-fold higher when compared with the Hypoxia group (*p* < 0.05), suggesting that reducing mitochondrial Drp1 levels may improve both mitochondrial morphology and mPTP opening after hypoxia (Fig. 1B). However, after treatment with CsA (10 μM) [14], a specific inhibitor of mPTP opening, only hypoxia-induced mPTP overopening was significantly improved (*p* < 0.05), but little effect was found on mitochondrial morphology after CsA treatment (Fig. 1B), which may be due to the inability of CsA on mitochondrial Drp1 levels (Fig. 1C). These results suggested that simply inhibiting mPTP opening without reducing Drp1 mitochondrial translocation may not affect the mitochondrial fission process after hypoxia. In addition, we also examined the profile of mitochondrial CytC release to reflect mPTP opening. Our results showed that reducing mitochondrial Drp1 levels with Mdivi-1 could significantly reduce mitochondrial CytC release (*p* < 0.05), indicating the effects of mitochondrial Drp1 on mPTP opening regulation and mPTP overopening may be downstream of Drp1 mitochondrial translocation after hypoxia.

To investigate whether cytoplasmic Drp1 has a similar regulatory effect on mPTP opening, we subjected cytoplasmic Drp1 overexpression to normal VSMC (Fig. S1) (Drp1 OE + Normal group) and found that the mitochondrial skeleton length was significantly shortened (*p* < 0.05), whereas the mPTP opening was not significantly reduced (*p* > 0.05) when compared with Vector + Normal group (Fig. 1E). The above results suggested that cytoplasmic Drp1 may only influence the mitochondrial fission process and had no effect on mPTP opening regulation. We further accelerated Drp1 mitochondrial translocation induced by hypoxia in Drp1 OE-treated VSMC (Drp1 OE + Hypoxia group) and found that the opening of mPTP was significantly decreased when compared with the Drp1 OE + Normal group (*p* < 0.05), and this effects could be reversed by supplementary CsA treatment (*p* < 0.05) (Fig. 1E). These results indicate that both cytoplasmic and mitochondrial Drp1 could affect mitochondrial morphology to some extent, but only mitochondrial Drp1 was involved in the regulation of mPTP opening after hypoxia.

### Mitochondrial Drp1 interacts with mPTP through BAX and PiC

**Fig. 2:**
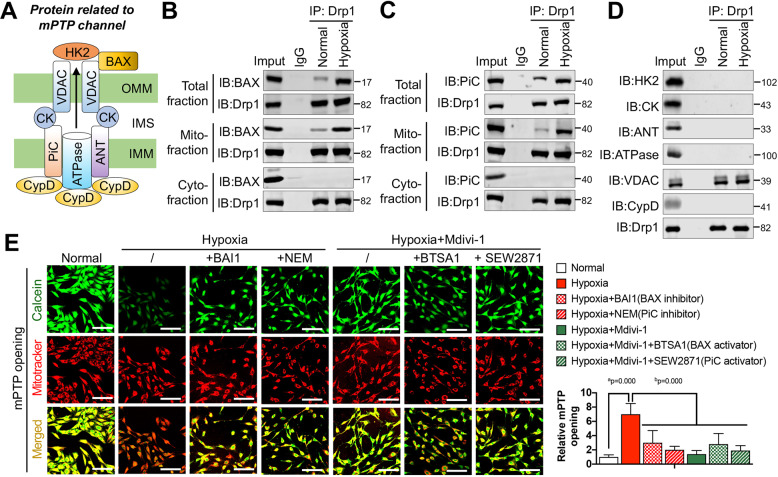
Mitochondrial Drp1 recognizes mPTP channels through binding with BAX and PiC after hypoxia. **A** The schematic diagram showing the proteins related to mPTP channel under current cognition. **B** Co-IP results for the combination of Drp1 and BAX in total, cytoplasmic and mitochondrial fractions of VSMCs after hypoxia. **C** Co-IP results for the combination of Drp1 and PiC in total, cytoplasmic and mitochondrial fractions of VSMCs after hypoxia. **D** Co-IP results for the combination of Drp1 and other mPTP related proteins, including HK2, CK, ANT, ATPase, VDAC and CypD, after hypoxia. **E** Representative confocal images of mPTP opening in VSMCs after hypoxia and Mdivi-1 (50 μM) treatment under intervening BAX or PiC (bar, 100μm). BAX inhibitor: BAX activation inhibitor 1 (BAI1, 5 μM), PiC inhibitor: N-Ethylmaleimide (NEM, 5 μM), BAX activator: BAX trigger site activator 1 (BTSA1, 15 μM), PiC activator: spingosine-1 phosphate receptor agonist (SEW2871, 15 nM). Relative mPTP opening ratio (fluoresce intensity of MitoTracker/Calcein) in each group was calculated by Image J (*n* = 5 samples/group). a: *P* < 0.05 compared with Normal group, b: *P* < 0.05 compared with Hypoxia group.

To understand the mechanism underlying the effects of mitochondrial Drp1 on mPTP opening after hypoxia, we first explored whether mitochondrial Drp1 interacts with mPTP after hypoxia. Previous studies [15–17] have shown that mPTP consists of various proteins spanning the inner and outer mitochondrial membranes. It consists of voltage-dependent anion channel (VDAC), Bcl-2-associated X protein (BAX), and hexokinase 2 (HK2) in the outer mitochondrial membrane; adenine nucleotide translocase (ANT), mitochondrial phosphate carrier (PiC), adenosine triphosphatase (ATPase) [18] and cyclophilin D (CypD) in the inner mitochondrial membrane; and creatine kinase (CK) between the inner and outer mitochondrial membranes [19] (Fig. 2A). We performed co-IP to screen the mPTP channel proteins for binding with Drp1 after hypoxia and found that more BAX and PiC bound to Drp1 in the hypoxia group than in the normal group (*p* < 0.05), and that this binding applies only to mitochondrial proteins (Fig. 2B, C). More VDAC also appeared to bind Drp1 after hypoxia, but the amount of VDAC bound to Drp1 in the hypoxia group did not significantly differ from that in the normal group (*p* > 0.05) (Fig. 2D). These results suggested that mitochondrial Drp1 may target and interact with mPTP after hypoxia by binding BAX and PiC.

To test our hypothesis, we performed Calcein fluorescence staining on hypoxia-induced VSMC treated with BAX activation inhibitor 1 (BAI1, a BAX inhibitor, 5 μM) and N-Ethylmaleimide (NEM, a PiC inhibitor, 5 μM) [20]. We found that mitochondrial Calcein fluorescence intensity increased to varying degrees after BAX or PiC inhibition in hypoxia-induced VSMC respectively (*p* < 0.05) (Fig. 2E), suggesting that the binding of mitochondrial Drp1 to BAX and PiC is important for mPTP overopening after hypoxia. Additionally, the BAX agonist BTSA1 (15 μM) and the PiC agonist SEW2871 (15 nM) [21] were unable to significantly affect mitochondrial Calcein fluorescence intensity on hypoxia-induced VSMC when the levels of mitochondrial Drp1 were reduced using Mdivi-1 (*p* > 0.05) (Fig. 2E), which further suggested that the interaction of mitochondrial Drp1 with BAX and PiC is indispensable to the mechanism underlying mPTP overopening in response to hypoxia.

### Mitochondrial Drp1 leads to mPTP overopening after hypoxia by promoting dissociation of HK2 from the mitochondrial membrane

**Fig. 3:**
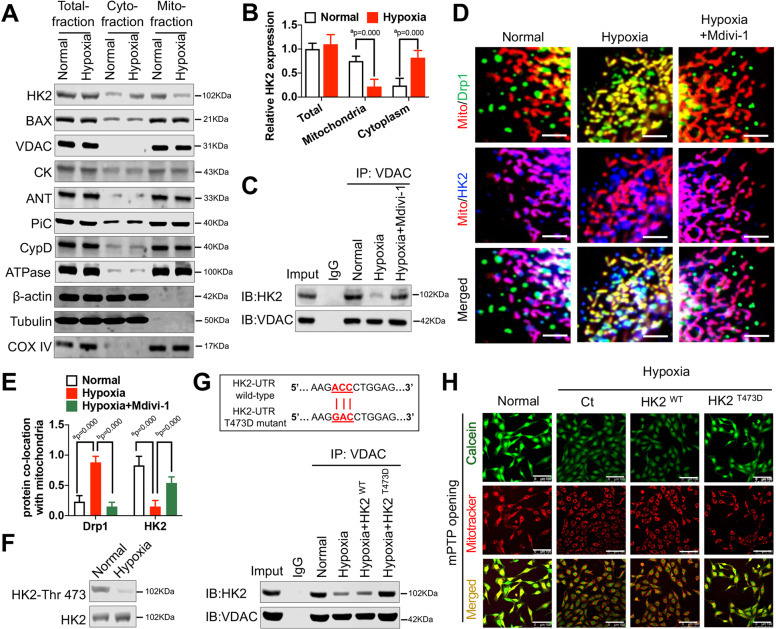
Mitochondrial Drp1 leads to excessive mPTP opening through inducing HK2 mitochondrial separation after hypoxia. **A** and **B** The expression and statistical analysis of all mPTP related proteins in total, cytoplasmic and mitochondrial fractions of VSMCs after hypoxia. β-actin, Tubulin, and COX IV were used as interior references of total, cytoplasmic and mitochondrial fractions. (*n* = 8 samples/group). **C** Co-IP results for the combination of HK2 and VDAC in hypoxia-induced VSMCs after Mdivi-1 (50 μM) treatment. **D** and **E** Representative immunofluorescence images and statistics analysis showing the co-location of Drp1 (green), HK2 (blue), and mitochondria (red) in hypoxia-induced VSMCs after Mdivi-1 (50 μM) treatment. (bar, 5 μm). **F** Western blot analysis showing the phosphorylation of HK2-Thr 473 in VSMCs after hypoxia. **G** Co-IP results for the combination of HK2 and VDAC in hypoxia-induced VSMCs after HK2 T473D mutation. **H** Representative confocal images of mPTP opening in hypoxia-induced VSMCs after HK2 T473D mutation. (bar, 100 μm). a: *P* < 0.05 compared with Normal group, b: *P* < 0.05 compared with Hypoxia group.

To determine whether the mechanism by which mitochondrial Drp1 regulates mPTP overopening is also related to an altered mPTP structure, we first examined the expression of all mPTP related proteins in different cellular components after hypoxia. Western blotting results showed that cytoplasmic HK2 expression was significantly elevated and mitochondrial HK2 expression was significantly reduced after hypoxia (*p* < 0.05) (Fig. 3A, B), suggesting that the HK2 may have dissociated from the outer mitochondrial membrane after hypoxia. We next performed co-IP to examine the binding of HK2 to VDAC, an adjacent mPTP related protein, after hypoxia. We found that the amount of VDAC-bound HK2 was significantly reduced after hypoxia. Meanwhile, inhibition of Drp1 mitochondrial translocation using Mdivi-1 raised the amount of VDAC-bound HK2, suggesting that the dissociation of HK2 from the mitochondrial membrane after hypoxia is related to the increase in mitochondrial Drp1 levels (Fig. 3C). Confocal images showed significantly elevated Drp1 and significantly reduced HK2 colocalization with the mitochondria after hypoxia (*p* < 0.05). After inhibition of Drp1 mitochondrial translocation using Mdivi-1, Drp1 colocalization with the mitochondria was significantly reduced, and HK2 colocalization with the mitochondria was significantly elevated (*p* < 0.05) (Fig. 3D, E). These results reflect the effect of mitochondrial Drp1 on HK2 dissociation from the mitochondria after hypoxia.

Further analysis revealed that the dissociation of HK2 from the mitochondrial membrane after hypoxia may result from HK2 inactivation. Western blotting results showed that HK2 Thr473 phosphorylation levels were significantly reduced after hypoxia (Fig. 3F). Further, an HK2 T473D substitution significantly attenuated the hypoxia-induced HK2-VDAC binding (Fig. 3G) and mPTP overopening (Fig. 3H). These results suggest that the mechanism through which mitochondrial Drp1 leads to mPTP overopening is mediated by the dissociation of HK2 from the mitochondrial membrane, which may be due to reduced HK2 phosphorylation.

### Mitochondrial Drp1 leads to HK2 dephosphorylation and mitochondrial dissociation after hypoxia by occluding the LRRK2 active site

**Fig. 4:**
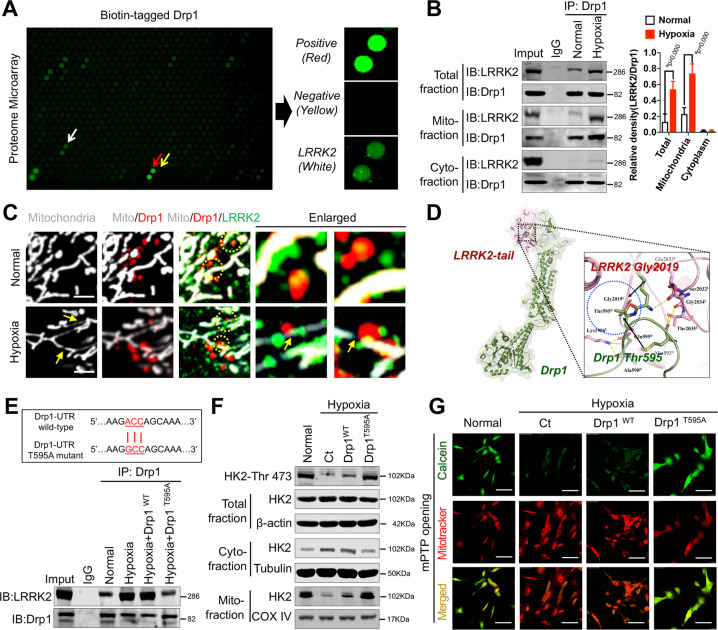
Mitochondrial Drp1 induces HK2 dephosphorylation through recruiting LRRK2 after hypoxia. **A** Human proteome microarray showing potential interacting proteins with Biotin-tagged Drp1 protein. Red arrow indicated the positive control (Biotin-BSA), yellow arrow indicated the negative control (BSA protein), and white arrow indicated LRRK2, our interested potential interacting proteins with Biotin-tagged Drp1 protein. **B** Co-IP results for the combination of Drp1 and LRRK2 in total, cytoplasmic and mitochondrial fractions of VSMCs after hypoxia. (*n* = 3 samples/group). **C** Representative immunofluorescence images showing the co-location of Drp1 (red), LRRK2 (green) and mitochondria (white) in VSMCs after hypoxia. (bar, 5 μm). **D** Molecular docking of Drp1 to LRRK2 in hypoxia conditions determined by GRAMM-X Protein-Protein Docking program. The blue dotted indicated the stable hydrogen bond between Drp1 Thr595 and LRRK2 Gly2019. **E** Co-IP results for the combination of Drp1 and LRRK2 in hypoxia-induced VSMCs after Drp1 T595A mutation. **F** The phosphorylation of HK2-Thr 473 and HK2 expression in total, cytoplasmic and mitochondrial fractions of hypoxia-induced VSMCs after Drp1 T595A mutation. β-actin, Tubulin and COX IV were used as interior references of total, cytoplasmic and mitochondrial fractions. **G** Representative confocal images of mPTP opening in hypoxia-induced VSMCs after Drp1 T595A mutation. (bar, 100 μm). a: *P* < 0.05 compared with Normal group.

To investigate how Drp1, which does not exhibit kinase and phosphatase activities, affects HK2 activity after hypoxia, we performed protein chip high-throughput sequencing of biotin-labeled Drp1 proteins to screen for kinases or phosphatases that potentially interact with Drp1 after hypoxia. The microarray results suggested an interaction between Drp1 and leucine-rich repeat serine/threonine-protein kinase 2 (LRRK2) after hypoxia (Fig. 4A). Co-IP results confirmed that the amount of LRRK2-bound mitochondrial Drp1 significantly increased after hypoxia (*p* < 0.05), and that the free cytoplasmic Drp1 hardly bound to LRRK2 (Fig. 4B). Similarly, confocal images suggested that mitochondrial Drp1 recruited large amounts of LRRK2 to the mitochondrial fission site after hypoxia, whereas there was little colocalization between cytoplasmic Drp1 and LRRK2 (Fig. 4C).

The GRAMM-X Protein–Protein Docking algorithm predicted hydrogen bonding between mitochondrial Drp1 and LRRK2 through a number of amino acid residues. The top three potential binding sites were Drp1 Thr595 with LRRK2 Gly2019, Drp1 Lys594 with LRRK2 Glu2033, and Drp1 Ala598 with LRRK2 Lys1906. Among these, the predicted hydrogen bond between Drp1 Thr595 and LRRK2 Gly2019 had the highest ZDOCK score of 1330.963 (Fig. 4D). To verify the specific binding site of mitochondrial Drp1 to LRRK2 after hypoxia and the effects of the Drp1–LRRK2 interaction on mPTP overopening, we introduced a T595A substitutions to Drp1. We found that post-hypoxia binding of Drp1 to LRRK2 was significantly attenuated by the T595A substitution in Drp1 (*p* < 0.05) (Fig. 4E). The Drp1 T595A substitution also led to significantly elevated HK2 Thr473 phosphorylation (*p* < 0.05), significantly reduced HK2 mitochondrial dissociation (*p* < 0.05) (Fig. 4F), and significantly reduced mPTP channel opening (*p* < 0.05) (Fig. 4G). These results suggest that, following hypoxia, mitochondrial Drp1 leads to reduced HK2 Thr473 phosphorylation and mitochondrial HK2 dissociation by recruiting LRRK2 and blocking the LRRK2 G2019 active site, ultimately leading to mPTP overopening.

## Discussion

**Fig. 5:**
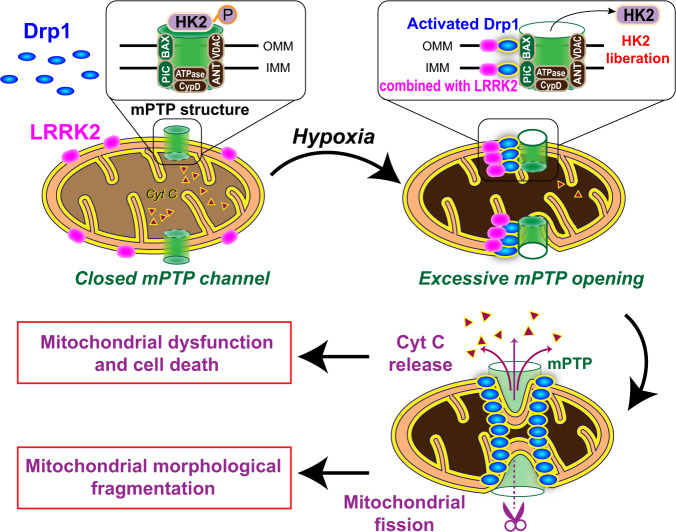
The schematic diagram showing the mechanisms of mitochondrial Drp1 recognizing and inducing excessive mPTP opening after hypoxia. Hypoxia-activated mitochondrial Drp1 firstly recognizes mPTP channels through binding with its structural proteins, BAX and PiC, then recruits LRRK2 to mitochondrial contraction or fission sites and blocks its kinase activity site (LRRK2 G2019), leading to the dephosphorylation and mitochondrial separation of mPTP related protein HK2, resulting in structural damage and excessive opening of mPTP channel after hypoxia.

In this study, we found that, in addition to regulating mitochondrial morphology through the classical mitochondrial fission-dependent pathway, activated Drp1 undergoing mitochondrial translocation further recognizes and induces excessive mPTP opening after hypoxia. Firstly, mitochondrial Drp1 maximumly recognizes mPTP channels by binding the mPTP structural proteins BAX and PiC. Then, mitochondrial Drp1 recruits LRRK2 to mitochondrial contraction or fission sites and then blocks the LRRK2 kinase active site. This then leads to HK2 dephosphorylation and its dissociation from the mitochondrial membrane, ultimately causing structural disruption and overopening of mPTP, ultimately causing structural disruption and overopening of mPTP, which aggravates mitochondrial and cellular dysfunction after hypoxia (Fig. 5).

The connection between mitochondrial morphology and mitochondrial function has been the focus of several studies in the field of mitochondrial regulation. The study of Han et al. [22] showed that hypoxia-induced ROS accumulation in cervical cancer cells promoted mitochondrial fission through the downregulation of Drp1 Ser637 phosphorylation. The study of Zhang et al. [23] showed that hypoxia-induced Drp1 Ser616 activation in pancreatic β-cells triggered the release of CytC and the activation of caspases, ultimately leading to pancreatic β-cell apoptosis. These studies suggest that Drp1 is a mechanochemical enzyme that is likely to play a role in the dual regulation of mitochondrial morphology and function after hypoxia. Consistent with our findings, a study on myocardial ischemia by Dhingra et al. [24] showed that Drp1 activation was accompanied by mPTP channel opening and reduced mitochondrial membrane potential after hypoxia. However, Dhingra et al. did not elucidate the direct effects and potential regulatory role of Drp1 on mPTP opening. Here, we have shown that, following hypoxia, mitochondrial Drp1 promotes mPTP overopening through interactions with BAX-PiC and LRRK2-HK2, which aggravate the mitochondrial injury and cell death. Our study highlights the role of Drp1 in the regulation of mitochondrial morphology and function and suggests a novel mechanism by which Drp1 participates in the regulation of mitochondrial homeostasis after hypoxia.

Previous studies [25, 26] have shown that Drp1 can interact with a variety of proteins, such as mitochondrial dynamics proteins (MiD49 and MiD51), the mitochondrial fission factor (MFF), and the mitochondrial fission 1 protein (Fis1). Most of these proteins are distributed on the outer mitochondrial membrane and act as receptors for Drp1 after its translocation to the mitochondria. These proteins are considered important for Drp1-mediated mitochondrial fission. Using a shock model, we have previously demonstrated that Drp1 binds BAX after ischemia and hypoxia, causing increased CytC release and caspase 3/9 activation, which promotes cell death after hypoxia [3]. In the present study, we showed that the Drp1-induced cell death was primarily related to the overopening of mPTP and that this was mediated by the interaction of Drp1 with BAX in the outer mitochondrial membrane and PiC in the inner mitochondrial membrane following hypoxia. Studies have shown that PiC plays an important role in ATP synthesis and can mediate the transport of inorganic phosphate from the mitochondrial inner membrane to the matrix [27, 28], and overexpression of PiC protein can induce apoptosis [29]. The study of Kwong et al. [30] showed that cardiac-specific PiC mutant mice had reduced sensitivity to mPTP opening and tolerated ischemic-hypoxic injury to some extent. However, a PiC mutation did not prevent mPTP opening. Here, we have elucidated a potential reason underlying these results. Although PiC is an mPTP related protein, it is only involved in the binding of mPTP to Drp1 and is not directly involved in the subsequent regulation of mPTP opening. Low levels of PiC may only reduce the interaction between mPTP and mitochondrial Drp1 and may only have a weak influence on mPTP opening after hypoxia.

Several factors, such as calcium overload [31], oxidative stress [32], abnormal pH, and increased inorganic phosphate, affect mPTP opening. In addition, mPTP exhibits the capacity to regulate its own structure [33]. ANT in the inner mitochondrial membrane portion of mPTP can control ATP to ADP conversion, and it can influence mPTP opening by altering mitochondrial energy metabolism [34]. Overopening of mPTP is induced when ATP and ADP bind the ANT substrate binding site on the cytoplasmic side, and overopening of mPTP is inhibited when ATP and ADP bind to the ANT substrate binding site on the matrix side [35]. VDAC in the outer mitochondrial membrane portion of mPTP can affect the sensitivity of mPTP to calcium [36]. The study of Lee et al. [37] showed that Drp1 binding to VDAC was significantly elevated in prostate cancer, which resulted in increased pyruvate transport to the mitochondria and affected mitochondrial metabolic processes such as oxidative phosphorylation and lipogenesis. In this study, we similarly found that Drp1 binds VDAC, but the Drp1-VDAC binding was not significantly altered after hypoxic injury. This may be related to the fact that Drp1 mainly undergoes changes in activation and translocation in acute diseases such as ischemic-hypoxic injury [4, 10], whereas Drp1 mainly shows increased expression in chronic diseases such as tumors and diabetes [37, 38], and these differences may have varying effects on Drp1 binding to target proteins. CypD is an mPTP structural protein located within the mitochondrial matrix; oxidative stress can increase the binding of CypD to ANT and PiC and promote mPTP overopening [39]. The study of Xiao et al. [40] showed that CypD promoted the phosphorylation and mitochondrial translocation of Drp1 in neurodegenerative diseases. Our results suggest that the effects of CypD on Drp1 may be related to the interaction between mPTP and Drp1.

HK2 has been recently proposed as an mPTP structural protein [41]. HK2 was previously thought to only affect glucose metabolism to glucose 6-phosphate [42]. Recent studies have found that glucose 6-phosphate accumulation and decreased pH after prolonged ischemia could affect mitochondrial HK2 dissociation [43], and that enhancing the binding of HK2 to the outer mitochondrial membrane alleviates myocardial injury caused by ischemia and hypoxia [41]. In tumor cells, Akt activation can induce phosphorylation of HK2 Thr473 and enhance HK2 binding to the outer mitochondrial membrane [44]. In cardiomyopathy, glycogen synthase kinase 3 beta (GSK3β)-induced HK2 dephosphorylation can cause mitochondrial HK2 liberation [45], indicating that an altered HK2 activity is a key factor for mitochondrial HK2 expression and translocation. The influence of Drp1 on HK2 mitochondrial translocation has been previously proposed in myocardial ischemia-reperfusion injury [41], but its mechanism was not elucidated. In this study, we found that the effect of mitochondrial Drp1 on HK2 activity and translocation after hypoxia was mainly related to the recruitment of LRRK2 by Drp1 and the kinase domain mutation. The key interaction occurred between Drp1 Thr595 and LRRK2 G2019. Thus, our findings provide a new target for intervention in mPTP channel overopening studies. An interaction between Drp1 and LRRK2 has also been previously observed in other models [46]. Abnormalities in LRRK2 cause F-actin hyperstabilization and Drp1 mislocalization, which induce neurotoxicity of the microtubule-binding protein Tau in Parkinson’s disease [47]. In addition, previous studies have shown that LRRK2 is important in the regulation of mitochondrial dynamics in hypoxia [48]. Recent studies [49] have shown that LRRK2 itself can regulate mitochondrial homeostasis, and LRRK2 can disrupt the interaction between Drp1 and Parkin on the mitochondrial membrane and inhibit mitophagy. Our study further indicates the regulatory role of LRRK2 in mitochondrial dynamics and functions, and that this regulatory process is closely related to Drp1.

This study has some limitations. Because there is no effective detection method for mPTP opening in vivo, we cannot verify our results in animal models. Next, our protein chip sequencing results suggested several potential Drp1-interacting proteins. The interaction of Drp1 with these other proteins and how they affect mitochondrial homeostasis after ischemic-hypoxic injury will have to be explored further. Lastly, this study continues our previous study on the mechanism of Drp1 in a mitochondrial mass imbalance in vascular tissues after ischemic-hypoxic injury. Whether the mechanism by which mitochondrial Drp1 recognizes and induces the excessive opening of mPTP in VSMC after hypoxia through BAX-PiC and LRRK2-HK2 also applies to other tissue cell types, such as cardiomyocytes and intestinal epithelial cells, will have to be explored in future studies.

## Conclusion

Here, we have shown the mechanisms underlying the effects of elevated mitochondrial Drp1 on mPTP opening after hypoxia. In particular, we report that the direct interaction of Drp1 with mPTP related proteins promotes mPTP destabilization through the LRRK2-mediated dissociation of HK2 from the mitochondrial membrane. Our results provide insights into the role of Drp1 on hypoxia-induced mitochondrial dysfunction and cell death and propose Drp1–mPTP pathway may be the effective potential targets for dual regulating mitochondrial morphology and functions in acute ischemic/ hypoxic injury.

## Supplementary information

Figure S1 legend

Figure S1

## Data Availability

All data in this study are available from the first or corresponding author upon reasonable request.
