# Supplementary material for: Mitochondrial Drp1 recognizes and induces excessive mPTP opening after hypoxia through BAX-PiC and LRRK2-HK2
Source: Cell Death Dis. 2021 Nov 5;12(11):1050. doi: 10.1038/s41419-021-04343-x (PMC8571301; doi:10.1038/s41419-021-04343-x)
Supplement: Supplementary file 1 — Figure S1 legend [file 41419_2021_4343_MOESM1_ESM.docx]

**Supplementary Materials**

**Figure S1**

Relative protein expression of Drp1 in total, cytoplasmic and mitochondrial fractions of Drp1-over expressed (Drp1 OE) VSMCs after hypoxia and CsA (10μM) treatment. β-actin, Tubulin and COX IV were used as interior references of total, cytoplasmic and mitochondrial fractions. (n = 8 samples/group).
