# Supplementary figures and images for: Mitochondrial Drp1 recognizes and induces excessive mPTP opening after hypoxia through BAX-PiC and LRRK2-HK2
Source: Cell Death Dis. 2021 Nov 5;12(11):1050. doi: 10.1038/s41419-021-04343-x (PMC8571301; doi:10.1038/s41419-021-04343-x)

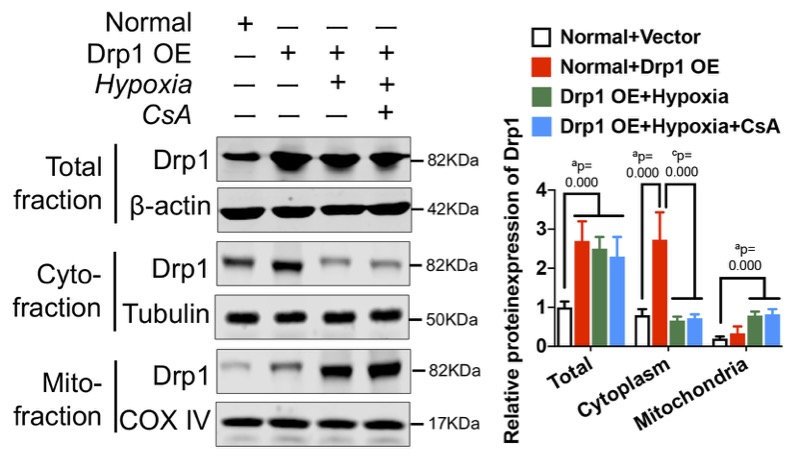

Supplement: Supplementary file 2 — Figure S1 [file 41419_2021_4343_MOESM2_ESM.jpg]
